# Hyperspectral Remote Sensing Image Classification Based on Maximum Overlap Pooling Convolutional Neural Network

**DOI:** 10.3390/s18103587

**Published:** 2018-10-22

**Authors:** Chenming Li, Simon X. Yang, Yao Yang, Hongmin Gao, Jia Zhao, Xiaoyu Qu, Yongchang Wang, Dan Yao, Jianbing Gao

**Affiliations:** 1 College of Computer and Information, Hohai University, Nanjing 211100, China; lichenming55@163.com (C.L.); rcyyang@hhu.edu.cn (Y.Y.); zhaojia925@163.com (J.Z.); quxiaoyu@hhu.edu.cn (X.Q.); wangyongchang@hhu.edu.cn (Y.W.); Yao__Dan@163.com (D.Y.); 15195891861@163.com (J.G.); 2 School of Engineering, University of Guelph, Guelph, ON N1G 2W1, Canada; syang@uoguelph.ca

**Keywords:** remote sensors, hyperspectral remote sensing image, image classification, convolution neural network

## Abstract

In a traditional convolutional neural network structure, pooling layers generally use an average pooling method: a non-overlapping pooling. However, this condition results in similarities in the extracted image features, especially for the hyperspectral images of a continuous spectrum, which makes it more difficult to extract image features with differences, and image detail features are easily lost. This result seriously affects the accuracy of image classification. Thus, a new overlapping pooling method is proposed, where maximum pooling is used in an improved convolutional neural network to avoid the fuzziness of average pooling. The step size used is smaller than the size of the pooling kernel to achieve overlapping and coverage between the outputs of the pooling layer. The dataset selected for this experiment was the Indian Pines dataset, collected by the airborne visible/infrared imaging spectrometer (AVIRIS) sensor. Experimental results show that using the improved convolutional neural network for remote sensing image classification can effectively improve the details of the image and obtain a high classification accuracy.

## 1. Introduction

Hyperspectral remote sensing imaging is one of the hottest issues in the field of remote sensing. Remote sensing refers to the non-contact, remote detection of the radiation and reflection characteristics of electromagnetic waves of objects by means of sensors [1]. Hyperspectral remote sensing images (HSI) are obtained by high-resolution optical sensors; these images generally consist of tens or even hundreds of different spectral bands of the same remote sensing target and can be viewed as a three-dimensional (3D) dataset [2]. Continuous data can be obtained spatially and spectrally. HSIs contain a large amount of data and can provide hundreds of continuous and subdivided spectral bands. Therefore, HSI has good application prospects.

The development of hyperspectral remote sensing technology mainly benefits from the development and maturity of imaging spectrum technology. So far, more than 40 sets of international aviation imaging spectroradiometer are in running state, including AVIRIS, developed by NASA’s jet laboratory, HYDICE, developed by the U.S. naval research laboratory, ROSIS, developed by the reflection imaging spectrometer in Germany, FTHSI, represented by the third-generation hyperspectral imager, and Hyperion, aboard the EO-1 earth observation satellite launched by the U.S. [3]. The development of imaging spectrometer in China is closely following the international development. For example, airborne imaging spectrometers PHI and OMIS [4] have been successfully developed in China. They can obtain spectral information of 224 and 128 continuous bands, respectively. PHI and OMIS show the advanced level of the Asian imaging spectrometers among the many high-light spectral imaging equipment independently developed by China. Therefore, it can be seen that the short-wave infrared hyperspectral camera is at the forefront of the international imaging spectrum research.

Most scholars initially used traditional processing methods, such as the support vector machine (SVM) [5], k nearest neighbor classification algorithm (KNN) [6], and the Bayesian network [7], for HSI to classify surficial objects. However, these classification results were not ideal. In recent years, deep learning has received a considerable research attention from scholars, such as the Deep Belief Nets (DBN) [8], Restricted Boltzmann Machine (RBM) [9] and Automatic encoder (AE) [10]. In particular, convolutional neural network (CNN) has been confirmed to exhibit an excellent image processing performance [11,12,13,14]. However, in the traditional CNN structure, pooling layers generally adopt average pooling and are non-overlapping pools [15]. This structure mainly refers to using a fixed-size sampling window in the pooling layer to perform an average pooling operation on all non-overlapping fixed-size regions in the convolutional layer and output corresponding feature maps. However, using non-overlapping average pooling tends to result in unclear and difficult-to-distinguish extracted image features and a serious loss of image detail features, thereby affecting the subsequent classification accuracy. To avoid this problem, many scholars have selected to adopt the largest pooling method. For example, Serre et al. applied two-dimensional (2D) maximum pooling for optimization [16], and Fu et al. proposed a 3D maximum pooling method [17]. However, these researchers did not observe the effect of the relationship between the step and pooling nuclear sizes on classification accuracy. That is, when a step size is greater than or equal to a pooling nuclear size, the experimental results are unsatisfactory, fine experimental results cannot be obtained, many details are overlooked, expected requirements are unsatisfied, and considerable HSI information cannot be exploited.

To solve the abovementioned problems, in this paper, an improved convolutional neural network structure was studied. Based on the Alexnet network, the pooling method was improved, in which the maximum pooling was adopted in the pooling layer to avoid the fuzzy effect of average pooling. In the maximum overlap pooling CNN, the step size was smaller than the size of the pooling kernel. Thus, the output of the pooling layer overlapped and covered to form an overlapping pool, thereby improving the details of the image and the classification accuracy. This study aims to propose an improved remote sensing image classification algorithm on the basis of CNN and to extract valuable feature information from this; experiments show that the proposed method is superior to the old one in performance. This work is critical to improve the classification accuracy of HSI.

## 2. Convolutional Neural Network

**Figure 1:**
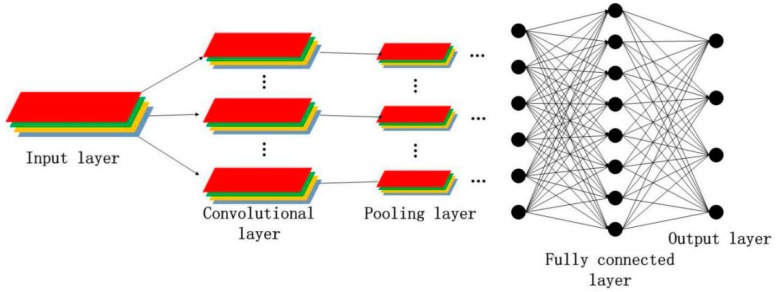
Typical model structure of a convolutional neural network (CNN).

The CNN is mainly composed of input, convolutional, pooling, fully-connected, and output layers [18]. Figure 1 illustrates a typical model structure of a CNN.

### 2.1. Convolutional Layer

The full connection of neurons between two adjacent layers is infeasible when the input of the neural networks is an HSI. The convolutional layer and neurons in the upper layer are connected in part through a local receptive field, because the full connection method disregards the spatial structure of an input image. That is, the neurons of the next layer are connected to a certain part of the neurons in the previous layer, and thus, indicate that the local features are extracted using the spatial structure of the input image. In addition, the convolutional layer reduces the number of model parameters by sharing weights and lessens the complexity of the network model. The convolutional layer in the CNN is crucial for feature extraction. The feature obtained by the local receptive field method has an invariance of translation, rotation, and scaling. The output of the convolutional layer is a feature map of the convolutional layer in the network depicted in Figure 1.

**Figure 2:**
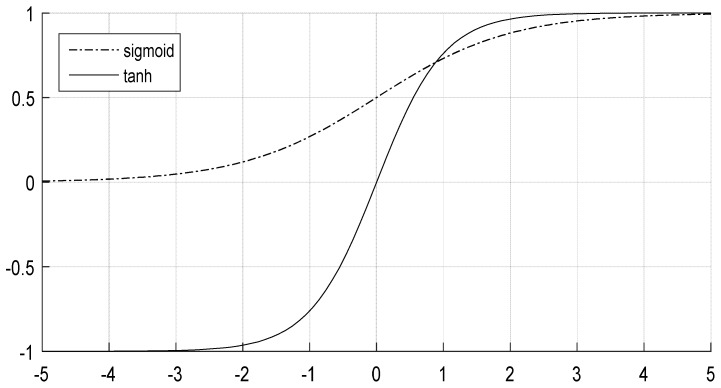
The curve of sigmoid function and tanh function.

Let the original image of the input of the CNN be P, then $F_{i}$ is used to denote the feature map of the *i*-th layer. A convolutional layer is assumed, and generation process can be described as follows:

If $F_{i}$ represents a convolutional layer, then the $F_{i}$ creation process can be defined as

(1) $$F_{i}=f(F_{i-1}⊗W_{i}+b_{i})\;$$

where $W_{i}$ represents the weight of the *i*-th layer convolution, $b_{i}$ represents the offset of the *i*-th layer, ⊗ represents the convolution of the *i*-th layer feature map using the convolution kernel, *f* represents the activation function, and $F_{i}$ represents the feature map of the *i*-th layer. In a conventional CNN, a saturated nonlinear function, such as a sigmoid or a tanh function, is generally used as an activation function, and the output value is mapped to (0, 1) or (−1, 1) through an activation function. The sigmoid function is expressed as

(2) $$f(x)=\frac{1}{1+e^{-x}}\;$$

and the tanh function is defined as

(3) $$f(x)=\frac{e^{x}-e^{-x}}{e^{x}+e^{-x}}\;$$

their curves are shown in Figure 2.

**Figure 3:**
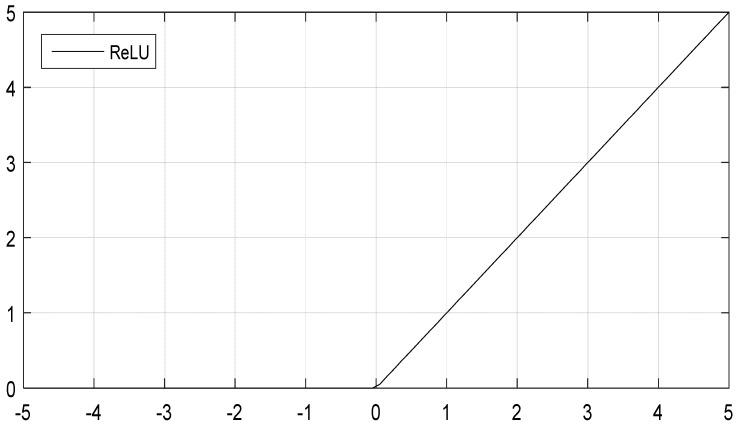
ReLU function curve.

However, a saturation nonlinear function easily leads to explosion or disappearance of a gradient, and the convergence is slow. Therefore, in the current CNN structure, an unsaturated nonlinear function similar to the rectified linear unit (ReLU) function [19] was used as an activation function of the convolutional layer, and ReLU function expression is *f*(*x*) = max(0, *x*). The curve is exhibited in Figure 3.

The ReLU can achieve sparse parameters through a simple thresholding activation function, and the training is faster than the sigmoid and tanh functions.

The convolutional layer extracts different features of the input image through different-sized convolutional kernels. An underlying convolutional layer mainly extracts low-level features, such as lines, edges, and corners, whereas a high-level convolutional layer extracts advanced features, such as clear semantic information, to improve the recognition accuracy.

### 2.2. Pooling Layer

The pooling layer is also called the downsampling layer [20]. This layer aims to achieve local averaging and sampling. Pooling not only reduces the eigenvector dimension and the number of parameters of a model but also reduces the sensitivity of the output features to factors, such as translation, rotation, and scaling, to prevent overfitting. The combination of the pooling and convolutional layers constitutes a two-time feature extraction structure, which strengthens the tolerance of a network model for distortion and enhances the robustness of the model [21]. 

Pooling methods include mean, maximum, and random pooling. Mean pooling mainly averages the pixels in a neighborhood and adopts a method for preserving the background information of an image to reduce the error caused by an estimation variance given the limited size of the neighborhood. Maximum pooling uses the maximum value of the pixels in the neighborhood to preserve image texture information and reduce the error of an estimated mean value offset caused by convolutional parameter errors. Random pooling between the mean and maximum pooling randomly selects the elements in a pooling feature layer by the size of a probability value; the probability for selecting a large-valued element is also high. In accordance with the pooling value, the pixel points are provided with a corresponding probability, after which downsampling is performed in accordance with the probability. 

According to the relevant theory, the error of feature extraction mainly comes from two aspects: (1) the variance of the estimated value increases due to the size of the neighborhood constraints; (2) the error of convolution layer parameters causes the deviation of the estimated mean. Generally speaking, average pooling can reduce the first error and preserve more background information of the image. Maximum pooling can reduce the second error and retain more texture information. Random pooling is between the two. By assigning probability to pixels according to their numerical values, and then sub-sampling according to the probability, it obeys the criterion of maximum pooling in the mean sense and approximate to the mean pooling in the local sense.

### 2.3. Fully Connected Layer

Several fully connected layers were added at the end of the CNN model after several convolutional and pooling layers. Each neuron in the fully connected layer was fully connected to all neurons in the previous layer, and the output value of the last fully connected layer was passed to the output layer that is classified using SoftMax logistic regression classifier [22].

## 3. Hyperspectral Image Classification Based on Maximum Overlap Pooling CNN

A new hyperspectral image classification based on maximum overlap pooling CNN was designed in this paper. This chapter mainly introduces the main structure of the CNN designed and the main contributions made.

### 3.1. Major Improvement Methods and Advantages

Scholars have slightly focused on the influence of the relative relationship between step and pooling nuclear sizes on the classification accuracy in previous works. Most scholars have opted to equalize step and pooling nuclear sizes during experiments. We observed that, if the pooling step is larger than the pooling kernel size, then the effect is close to the situation where the step and pooling kernel sizes are equal. However, if the pooling step size is smaller than the pooling kernel size, then the CNN classification accuracy will be improved. We considered that these results are due to the outputs of the pooling layer will overlap and cover one another and form overlapping pools, thereby improving the details of the image and the classification accuracy.

We used this method to design a maximum overlap pooling CNN in which the pooling layer used the maximum pooling, and the step size was smaller than the pooling kernel size. Thus, the outputs of the pooling layers overlapped and covered one another and formed overlapping pools. Therefore, the details of the image were improved, and favorable experimental results were obtained.

### 3.2. Training Model Design

The CNN training process is mainly divided into two phases. The first stage is the forward propagation stage, consisting of:

(1) Select training samples.

(2) Randomly initialize weights, offsets, and error thresholds, and set a learning rate. The learning rate will affect the weight adjustment range. An excessive learning rate will cause the adjustment of the weights to omit the optimal value and the divergence of the network. A too small learning rate will cause the model to fall into the local optimal problem. We must initialize the learning rate on the basis of prior knowledge, analyze specific problems, and set the optimal learning rate. 

(3) Select a sample vector from the training sample, and input it into the network. The input vector enters the model from the input layer, trains the vector gradually to the output layer, and multiplies the input vector and the weight matrix in layers to obtain the output.

The second stage is the backpropagation stage [23]: 

(1) Calculate the error between the actual and the expected output values of a single sample vector. 

(2) In accordance with minimization error method, the error value calculated in Step (1) is propagated consecutively in layers to adjust the weight item and offset term. 

(3) Compare the network error value and error threshold after adjusting the weights. If the error value is less than the threshold, then proceed to the next step. If the error value is greater than the threshold, then the network model has not reached the expected goal and must proceed to Step (3) of the first stage to continue training.

(4) The relative ideal CNN is learned after the training, and the network parameters in the steady state are saved [24].

### 3.3. Classification Steps

**Figure 4:**
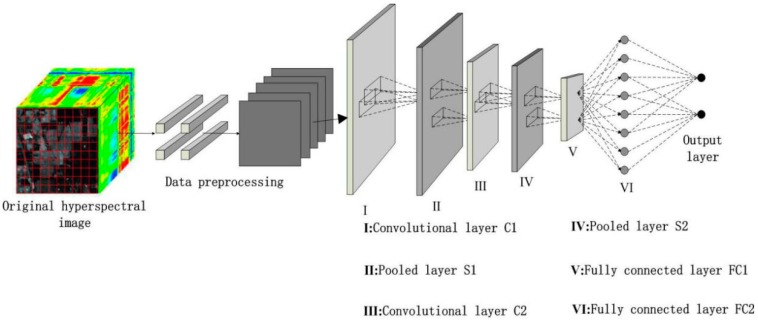
Image classification framework based on CNN.

This study used the concept of the LeNet-5 model [25] in designing an HSI classification model on the basis of the CNN, as displayed in Figure 4. The model consists of an input layer, two convolutional layers (C), two pooling layers (S), two full-attachment layers (FC), and a SoftMax regression output layer [26]. Among these layers, the preprocessing step completes the extraction of samples, normalizes input samples, and selects a 14 × 14 pixel window as the input sample of the model. The output section of the convolutional layer used the ReLU activation function to prevent gradient diffusion. The pooling layer used the maximum overlap pooling, which eliminated the requirement for additional processing of the raw image input to the CNN. The maximum overlap pooling method after each convolution of the original image was used to reduce the dimension of the convolution product and reduce the image size. Stochastic gradient descent method was used to optimize the weights of the network, and weight attenuation method [27,28] was also adopted.

The specific learning steps for HSI classification based on the maximum overlap pooling CNN framework are as follows:

(1) Input layer: The original data undergoes dimension reduction processing to extract a 14 × 14 pixel sample to ensure that the input of the model satisfies the requirements. Image classification refers to the classification of each pixel in accordance with a specific rule or algorithm based on the brightness, spatial characteristics, or other information of an image. In training a CNN, the convolution kernel convolutes each input to extract spatial structural features. A small block containing 145 × 145 pixels is selected as a sample centered on each pixel of the HSI to maintain the consistency with the input of the CNN; furthermore, each of the small blocks contains the spectral and spatial structure information of a specified pixel [29]. 

(2) Convolutional layer C1: The input pictures of the input layer are convolved with six 5 × 5 convolution kernels to obtain six 7 × 7 2D feature maps. The result is output to the next layer after multiplying the ReLU activation function and adding the offset. The size of the convolution kernel significantly influences the classification accuracy. If the convolution kernel is small, then local features cannot be effectively extracted; if the convolution kernel is large, then ideal characteristics cannot be obtained.

(3) Pooling layer S1: A 3 × 3 pixel sampling window is used through the maximum overlap pooling to perform the maximum pooling operation on all 2 × 2 areas in C1 and output six 4 × 4 pixel feature maps. The maximum overlap pooling CNN uses the maximum pooling than the average pooling commonly used in the traditional CNN to avoid the feature blurring caused by the average pooling. Moreover, the maximum overlap pooling CNN sets a smaller step size than the size of the pooling kernel; thus, the outputs of the pooling layer overlap and cover one another, thereby enhancing the details of the image. 

(4) Convolutional layer C2: An S1 output picture is convoluted using 5 × 5 convolution kernels to obtain 16 4 × 4 pixel 2D feature maps. The result is output to the next layer after multiplying the ReLU activation function and adding the offset.

(5) Pooling layer S2: A 3 × 3 pixel sampling window is used through the maximum overlap pooling to perform the maximum pooling operation on all 2 × 2 areas in C2 and output 16 2 × 2 pixel feature maps. Maximum pooling is still used, and the pooling step size is set smaller than the pooling kernel size to overlap and cover between the pooling layer outputs, thereby resulting in enhanced details.

(6) Fully connected layer FC1: The number of neurons of the fully connected layer FC1 is set to 120, and the ReLU function is used as an activation function. The number of output neurons is 120. 

(7) Fully connected layer FC2: The number of neurons in the fully connected layer FC2 is set to 84, and the ReLU function is selected as the activation function. The number of output neurons is 84. 

(8) Output layer: The number of output neurons is related to the number of categories in the input image. The experimental data has 16 types of ground objects. Thus, the number of output neuron nodes is set to 16. 

(9) The forward propagation network structure is designed, and the backpropagation algorithm is used to optimize the network parameters.

(10) The trained CNN model is used to verify the classification of the input test samples.

**Figure 5:**
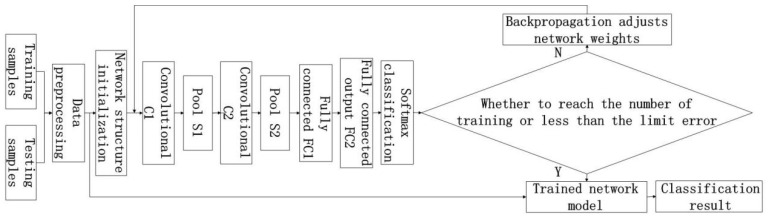
Classification flow chart of CNN hyperspectral remote sensing imaging (HSI).

The HSI classification flowchart based on the CNN is presented in Figure 5.

## 4. Experiments and Results Analysis

### 4.1. Experimental Environment

This study uses Google’s TensorFlow deep learning framework. TensorFlow supports multiple GPUs and distributed operations, supports different hardware platforms such as PCs and mobile phones, and has the advantages of an open source code and an active community. These advantages provide favorable accuracy and scalability for the experiments in this study.

This method was applied to actual HSI classification to validate the proposed method effectively, and simulation experiments were conducted. We used Intel Core i7 Quad-Core processor clocked at 2.50 GHz with 8 GB memory. We selected the 64-bits Windows 10 operating system, TensorFlow deep learning framework, and Python 2.7 as the development environment. We also utilized the following tools: MultiSpecWin64, MATLAB R2015b, and JetBrains PyCharm ×64.

In order to reduce the experimental error, the experimental results in this paper were obtained from the average of five experiments. Two data sets were adopted, namely, the Indian Pines dataset and Salinas dataset, as follows:

### 4.2. Experimental Data

With the development of sensor technology, the resolution of remote sensing image is getting higher and higher, which provides a strong support for remote sensing image classification. Nowadays, the progress of sensor technology is of great significance to the remote sensing field. Due to the development of sensor technology, the Indian Pines dataset and Salinas dataset adopted in this paper have higher resolution. The data in the Indian Pines dataset and Salinas dataset were all collected by an airborne visible/infrared imaging spectrometer (AVIRIS) sensor. AVIRIS was flown for the first time in 1986 (first airborne images), obtained its first science data in 1987, and has been fully operational since 1989. In June/July 1991, the instrument was flown over numerous European test sites in the framework of EMAC (European Multi-Sensor Airborne Campaign). AVIRIS uses scanning optics and a group of four spectrometers to image a 677 pixel swath width simultaneously in 224 contiguous spectral bands. A spatial image is built up through the scanner motion, which defines an image line 677 pixels wide perpendicular to the aircraft direction, and through the aircraft motion, which defines the length of the image frame. The sensor is an optomechanical whiskbroom scanner (12 Hz) that uses line arrays of detectors to image a 677 pixel-wide swath in 224 contiguous bands (four grating spectrometers). The spectral range is 360–2500 nm with a total of 224 bands [30]. 

The Indian Pines dataset of AVIRIS mainly covers the entire northwestern part of Indiana, USA. This dataset was derived from this website (http://www.ehu.eus/ccwintco/index.php?title=Hyperspectral_Remote_Sensing_Scenes). Its original image size was 145 × 145 pixels, with a spatial resolution of 20 m. The dataset contains 220 bands and 16 ground object categories, covering a spectral range of 0.2–2.4 phenotypes, with a spectral resolution of 10 nm. However, since the bands 104–108, 150–163, and 220 cannot be reflected by water, we generally used the remaining 200 bands after eliminating these 20 bands as the object of study. The number of different types of ground objects is shown in Table 1.

**Figure 6:**
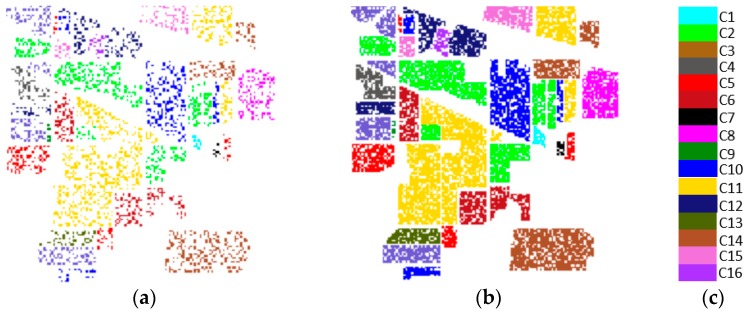
(**a**) Training sample; (**b**) Test sample; (**c**) Tag block.

Partial bands were deleted on the Indian Pines dataset to facilitate the conversion of space-spectral information of an HSI to a gray image with the same height and width. The (CVIE, Coefficient of Variation for Interclass)^2^/CVIA (Coefficient of Variation for Interclass) minimum 104–109, 149–164, 219, and 220 bands (for a total of 24 bands) were excluded, and the remaining 196 bands were retained. In addition, the 24 bands rejected by this method include the largest 20 bands that were affected by water and air noise in this dataset, that is, the 104–108, 150–163, and 220 bands. This result effectively enhances the reliability of the data and significantly reduces interference factors. The training and test samples obtained by pretreatment are shown in Figure 6. Table 1 shows the number of samples from the Indian Pines dataset.

The Salinas dataset of AVIRIS mainly covers the Salinas Valley. This dataset is derived from the same website as Indian Pines dataset. Its original image size was 512 × 217 pixels, and the spatial resolution was 3.5 m. The dataset contains 204 bands and 16 ground object categories. The number of different types of ground objects is shown in Table 2. 

**Figure 7:**
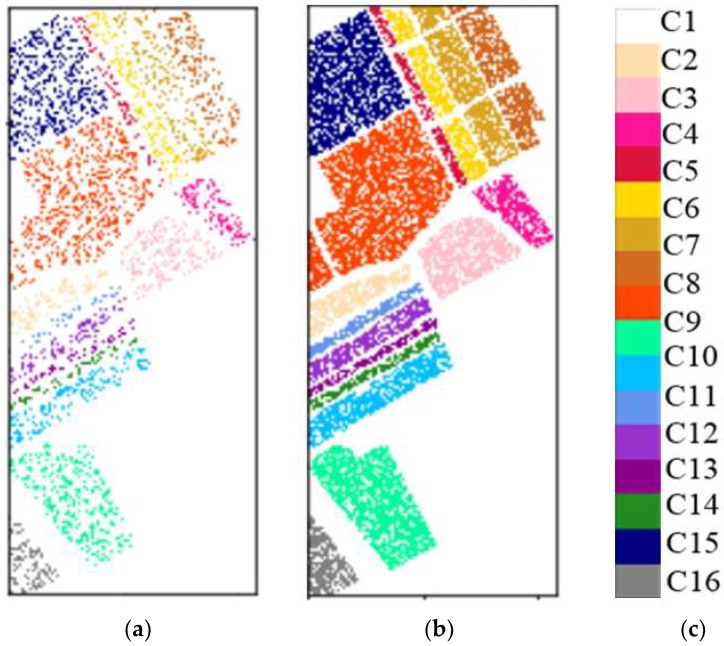
(**a**) Training sample; (**b**) Test sample; (**c**) Tag block.

Both of the two experimental data included 16 ground object categories. From all datasets, 25% were selected randomly as training samples, and the remaining 75% were used as test samples. The training and test samples obtained by pretreatment are shown in Figure 7. Table 2 shows the number of samples from the Salinas dataset.

### 4.3. Classification Results and Analysis

On the basis of the traditional and maximum overlap pooling CNNs, two kinds of CNN models were designed and used in this study to classify HSIs. The two methods were compared with the network-in-network (NIN) classification methods for HSIs. The network parameters of the traditional and maximum overlap pooling CNNs designed in this study are listed in Table 3 and Table 4.

#### 4.3.1. Comparison of Convergence Rates

**Figure 8:**
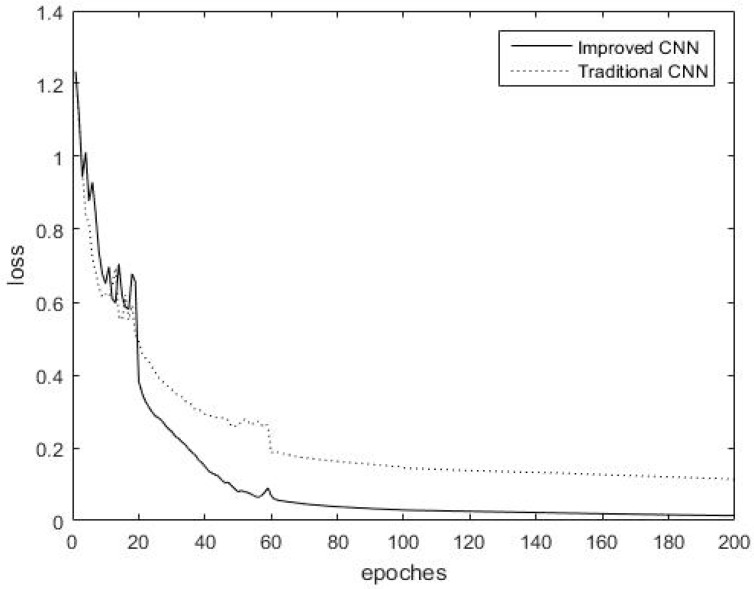
Training error of traditional CNN and maximum overlap pooling CNN iteration in the Indian Pines dataset.

All experiments in this paper were carried out under the same experimental environment. The variation of the training error with the increase in the number of iterations is exhibited in Figure 8 when two kinds of CNN are applied to the Indies Pines dataset.

Figure 8 displays that the training loss during training probably stabilized after 80 iterations in the Indian Pines dataset. Clearly, the maximum overlap pooling CNN converges more quickly than the traditional CNN during training. The maximum overlap pooling CNN may converge to the final loss accuracy of the traditional CNN approximately at the 50th iteration, which is nearly half of the time required by the traditional CNN. The maximum overlap pooling CNN, which has a lower training loss accuracy than the traditional CNN, can achieve better training results and fully learn the characteristics of the images. The maximum overlap pooling CNN demonstrates advantages over the traditional CNN in terms of training loss, with faster convergence speed and higher accuracy.

**Figure 9:**
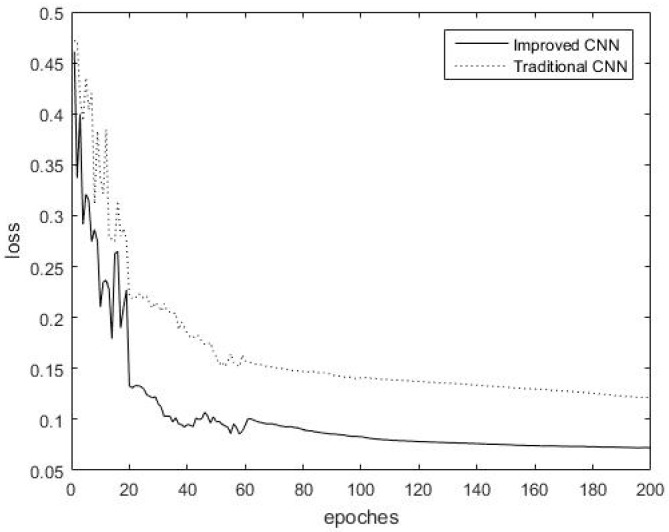
Training error of traditional CNN and maximum overlap pooling CNN iteration in the Salinas dataset.

Figure 9 displays that the training loss during training probably stabilized after 80 iterations in the Salinas dataset. Clearly, the maximum overlap pooling CNN converges more quickly than the traditional CNN during training. The maximum overlap pooling CNN may converge to the final loss accuracy of the traditional CNN approximately at the 30th iteration, which is less than half of the time required by the traditional CNN. The maximum overlap pooling CNN, which has a lower training loss accuracy than the traditional CNN, can achieve better training results and fully learn the characteristics of the images. The maximum overlap pooling CNN demonstrates advantages over the traditional CNN in terms of training loss, with faster convergence speed and higher accuracy.

#### 4.3.2. Comparison of Time and Classification Accuracies

Experiments were performed to verify the performance of the different methods in terms of accuracy. The experimental results where the Indian Pines dataset was used are summarized in Table 5, and the experimental results where the Salinas dataset was used are summarized in Table 6.

**Figure 10:**
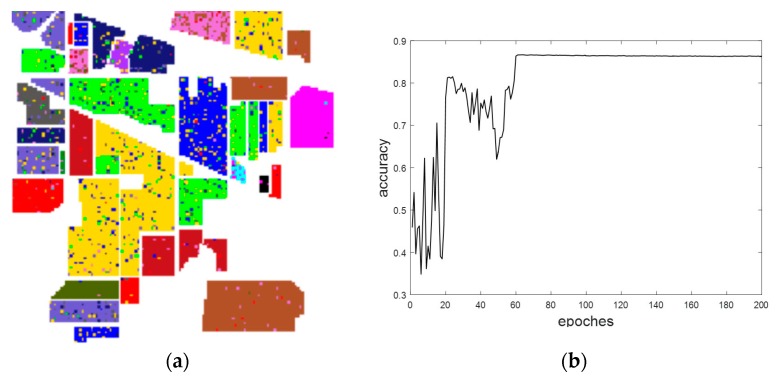
(**a**) Traditional CNN classification results. (**b**) Traditional CNN classification accuracy results.

**Figure 11:**
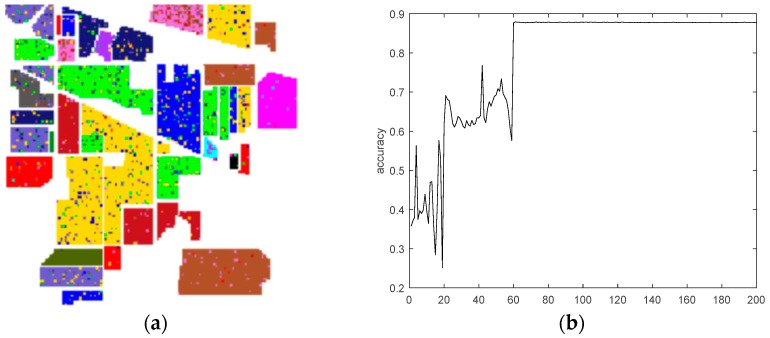
(**a**) Maximum overlapping pooling CNN classification results. (**b**) Maximum overlapping pooling CNN classification accuracy results.

Figure 10 demonstrates the results of the final classification accuracy based on the traditional CNN that used the Indian Pines dataset. Figure 11 exhibits the results of the final classification accuracy based on the maximum overlap pooling CNN that used the Indian Pines dataset.

**Figure 12:**
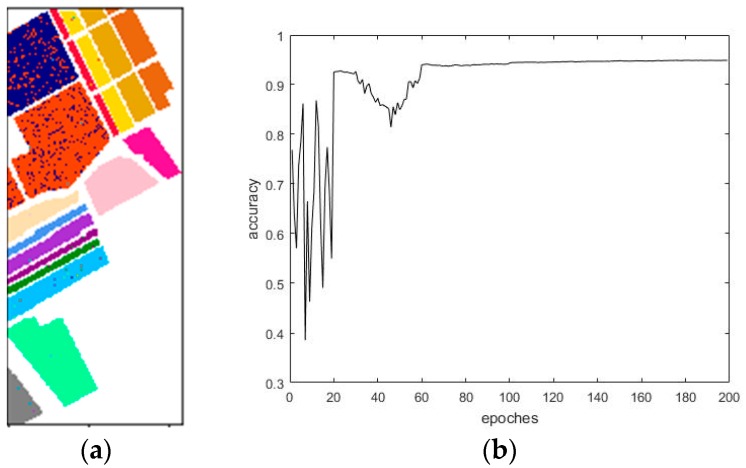
(**a**) Traditional CNN classification results. (**b**) Traditional CNN classification accuracy results.

**Figure 13:**
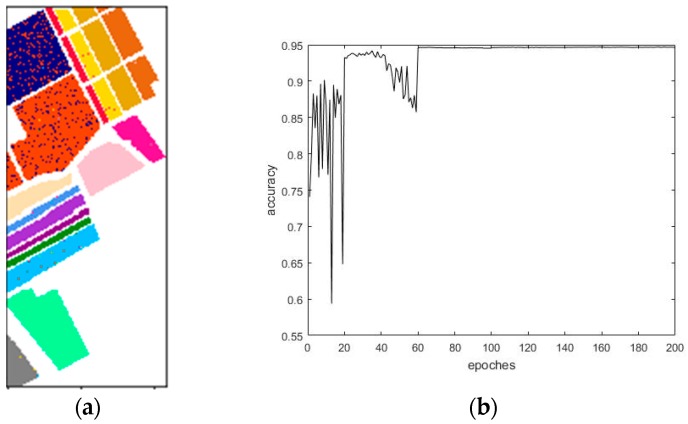
(**a**) Maximum overlapping pooling CNN classification results. (**b**) Maximum overlapping pooling CNN classification accuracy results.

Figure 12 demonstrates the results of the final classification accuracy based on the traditional CNN that used the Salinas dataset. Figure 13 exhibits the results of the final classification accuracy based on the maximum overlap pooling CNN that used the Salinas dataset.

Table 5 displays that, in the time accuracy analysis, the training and classification times when using the traditional CNN was the shortest, at only 114.60 s. The Densenet training recorded the longest time of 124.20 s. The classification time of the maximum overlap pooling CNN was 118.80 s has exhibited no obvious increase compared with the traditional CNN. Therefore, if the time accuracy is considered, the traditional CNN, Densenet, and maximum overlap pooling CNN method can be used.

The overall classification accuracy value reached 85.12%, the average accuracy reached 84.96%, the Kappa coefficient value was 0.8302, and the classification effect was poor. The analysis of the classification accuracy indicates that the overall classification accuracy reached 85.92%, the average accuracy reached 82.52%, the Kappa coefficient was 0.8397, and the classification effect was normal when the Densenet training was used. The classification accuracy reached 88.73%, the average accuracy reached 87.62%, the Kappa coefficient was 0.8714, and the accuracy was relatively favorable when the maximum overlap pooling CNN was used. The classification accuracy value is acceptable when the overall accuracy was higher than 85%, and the Kappa coefficient was more than 0.8. Therefore, if the classification accuracy is used as the evaluation basis, then the methods in the experiment all satisfy the requirements.

As can be seen from Table 6, from the time accuracy analysis, the training, and classification time of traditional convolution neural network training was the shortest, which only needed to be 584.40 s. The time required for Densenet training was 609 s. The classification time of the improved convolution neural network was 615.00 s. Compared to the traditional convolution neural network, the classification time did not increase significantly. Therefore, the traditional convolution neural network, Densenet and the improved convolution neural network method can be realized on the basis of time accuracy.

From the classification accuracy analysis and the training conducted by the traditional convolutional neural network, the overall classification accuracy reached 93.75%, the average accuracy reached 97.22%, the Kappa coefficient value was 0.9303, and the classification effect was poor; in the training conducted by Densenet, the overall classification accuracy reached 94.35%, the average accuracy reached 97.18%, the Kappa coefficient was 0.9372, and the classification effect was medium. Using the improved convolutional neural network classification training, the overall classification accuracy reached 94.76%, the average accuracy reached 97.45%, the Kappa coefficient was 0.9416, and the accuracy performance was relatively good. The overall accuracy of these three methods is above 93% and the Kappa coefficient is above 0.93. Therefore, if the classification accuracy is used as the evaluation basis, the methods in this experiment have met the requirements. Table 7 presents the confusion matrix of the traditional CNN classification that used the Indian Pines dataset, and Table 8 displays the corresponding mapping accuracy for when the Indian Pines dataset was used.

From the results in Table 7 and Table 8, we can conclude that the traditional CNN has achieved a favorable classification effect for the Indian Pines dataset. That is, there are 159 pixels in the 13th place category (Wheat) and 954 pixels in the 14th category (Woods) that have higher classification accuracy, achieving 98.74% and 97.48% in classification accuracy. The types of ground categories that were misclassified are mainly the first land category (Alfalfa) and the fourth land category (Corn), mainly because the total number of pixels in the two land categories was relatively small.

Table 9 lists the confusion matrix of the maximum overlap pooling CNN classifications that used the Indian Pines dataset. Table 10 summarizes the corresponding classification accuracy for when the Indian Pines dataset was used.

From the results provided in Table 9 and Table 10, we can conclude that the maximum overlap pooling CNN for the Indian Pines dataset achieves an improved classification effect. Among the result, the accuracy of the seventh land object type (Pasture-mowed) and the ninth land object type (Oats) reached 100.00%; thus, these land object types are not representative, because the total number of pixels was small. The total number of pixels in the eighth land category (Hay-windrowed) was high, and the accuracy is 99.17%. The types of ground objects that were mainly misclassified are the first floor object category (Alfalfa) and the fifteenth floor class (Building-trees).

Table 11 presents the confusion matrix of the traditional CNN classification that used the Salinas dataset. Table 12 displays the corresponding mapping accuracy for when the Salinas dataset was used.

From Table 11 and Table 12, it can be concluded that for the Salinas dataset, CNN obtained a good classification effect. In which the classification accuracy of most ground objects was higher, reaching above 96%. Ground objects category 5, Fallow_smooth, category 8, Grapes_untrained, and category 15, Vinyard_untrained, were mainly misclassified; it is believed that this was caused by the geographical proximity of these three types of ground objects and their similar spectra.

Table 13 lists the confusion matrix of the maximum overlap pooling CNN classifications that used the Salinas dataset. Table 14 summarizes the corresponding classification accuracy for when the Salinas dataset was used.

From Table 13 and Table 14, it can be concluded that for the Salinas data set, the improved CNN achieved better classification effect. The classification accuracy of most ground objects was higher, reaching over 97%. Ground objects category 8, Grapes_untrained, category 15, Vinyard_untrained, were mainly misclassified; it is believed that this was caused by the geographical proximity of these two types of ground objects and their similar spectra.

Based on the above experimental data, the maximum overlap pooling CNN has a high classification accuracy, which also achieves an ideal classification effect, and the training network model consumes less time.

## 5. Conclusions

This study proposes a framework for classifying the maximum overlap pooling CNN of HSI, which improve the pooling layer. The maximum overlap pooling CNN classification method was compared with the traditional CNN through experimental simulation. It can be concluded from the experimental results that the improved convolutional neural network is faster in loss convergence than traditional convolution in training, and that the training loss accuracy is lower, which can achieve a better training effect. Referring to the main results given in the experimental results section, the maximum overlap pooling CNN has a high classification accuracy, which also achieves an ideal classification effect, and the training network model consumes less time. Therefore, we conclude that the maximum overlap pooling CNN model has less training error, and the improved algorithm has a better effect on improving the classification accuracy of HSI and network convergence. The pooling layer can still be improved during the experiment, and further research on the improvement method will be conducted in the future.

## Figures and Tables

**Table 1:** Indian Pines dataset ground object type situation.

Label	Name	Number of Samples
C1	Alfalfa	46
C2	Corn-notill	1428
C3	Corn-mintill	830
C4	Corn	237
C5	Grass-pasture	483
C6	Grass-trees	730
C7	Grass-pasture-mowed	28
C8	Hay-windrowed	478
C9	Oats	20
C10	Soybean-notill	972
C11	Soybean-mintill	2455
C12	Soybean-clean	593
C13	Wheat	205
C14	Woods	1265
C15	Buildings-Grass-Trees-Drives	386
C16	Stone-Steel-Towers	93
Total		10,249

**Table 2:** Salinas dataset ground object type situation.

Label	Name	Number of Samples
C1	Brocoli_green_weeds_1	2009
C2	Brocoli_green_weeds_2	3726
C3	Fallow	1976
C4	Fallow_rough_plow	1394
C5	Fallow_smooth	2678
C6	Stubble	3959
C7	Celery	3579
C8	Grapes_untrained	11,271
C9	Soil_vinyard_develop	6203
C10	Corn_senesced_green_weeds	3278
C11	Lettuce_romaine_4wk	1068
C12	Lettuce_romaine_5wk	1927
C13	Lettuce_romaine_6wk	916
C14	Lettuce_romaine_7wk	1070
C15	Vinyard_untrained	7268
C16	Vinyard_vertical_trellis	1807
Total		54,129

**Table 3:** Traditional CNN parameter table.

Number of Layers	Species	Number of Output Features	Size of Output Features	Convolution Kernel Size
0	Input layer	1	14 × 14	/
1	Convolutional layer C1	6	7 × 7	5 × 5
2	Maximum pooling layer S1	6	4 × 4	2 × 2
3	Convolutional layer C2	16	4 × 4	5 × 5
4	Maximum pooling layer S2	16	2 × 2	2 × 2
5	Fully connected layer FC1	1	120	/
6	Fully connected layer FC2	1	84	/

**Table 4:** Maximum overlap pooling CNN parameter table.

Number of Layers	Species	Number of Output Features	Size of Output Features	Convolution Kernel Size
0	Input layer	1	14 × 14	/
1	Convolutional layer C1	6	7 × 7	5 × 5
2	Maximum pooling layer S1	6	4 × 4	3 × 3
3	Convolutional layer C2	16	4 × 4	5 × 5
4	Maximum pooling layer S2	16	2 × 2	3 × 3
5	Fully connected layer FC1	1	120	/
6	Fully connected layer FC2	1	84	/

**Table 5:** Convergence time and accuracy of different classification methods used Indian Pines dataset.

Method	Time/s	Kappa Coefficient	Overall Accuracy	Average Accuracy
Traditional CNN	114.60	0.8302	85.12%	84.96%
Densenet	124.20	0.8397	85.92%	82.52%
Maximum overlap pooling CNN	118.80	0.8714	88.73%	87.62%

**Table 6:** Convergence time and accuracy of different classification methods used Salinas dataset.

Method	Time/s	Kappa Coefficient	Overall Accuracy	Average Accuracy
Traditional CNN	584.40	0.9303	93.75%	97.22%
Densenet	609.00	0.9372	94.35%	97.18%
Maximum overlap pooling CNN	615.00	0.9416	94.76%	97.45%

**Table 7:** Confusion matrix for traditional CNN classification used Indian Pines dataset.

Category	1	2	3	4	5	6	7	8	9	10	11	12	13	14	15	16
1	20	0	0	0	4	0	0	9	0	0	1	1	0	0	1	0
2	0	913	34	23	0	0	0	0	2	18	84	9	0	0	0	0
3	0	9	518	40	0	0	0	0	1	2	24	16	0	0	1	0
4	0	5	18	033	0	4	0	3	1	2	4	3	0	0	0	0
5	3	5	1	2	326	3	0	0	0	0	5	3	0	1	1	0
6	0	0	0	0	0	523	0	0	0	0	3	0	0	4	12	0
7	0	0	0	0	0	0	19	1	0	0	1	0	0	0	0	0
8	11	0	0	0	2	0	0	350	0	0	0	0	0	0	0	0
9	0	0	0	0	0	0	0	0	11	0	0	0	0	0	1	0
10	0	23	3	1	0	3	0	0	0	608	88	2	0	0	1	0
11	0	70	106	6	0	0	0	0	0	30	1587	18	1	0	11	0
12	0	7	33	15	0	0	0	1	0	4	28	366	0	0	2	1
13	0	0	1	0	0	0	0	0	1	0	0	0	157	0	0	0
14	0	0	0	1	7	1	0	0	0	0	0	0	1	930	14	0
15	0	0	2	0	9	18	0	0	3	1	3	0	1	87	176	1
16	0	3	1	0	0	0	0	0	0	0	3	1	0	0	1	58

**Table 8:** Statistics of traditional CNN classification chart accuracy used Indian Pines dataset.

No.	Ground Category	Total Number of Pixels	Correct Classification	Classification Accuracy
1	Alfalfa	36	20	55.56%
2	Corn-notill	1083	913	84.30%
3	Corn-min	611	518	84.78%
4	Corn	73	33	45.21%
5	Grass/Pasture	350	326	93.14%
6	Grass/Trees	542	523	96.49%
7	Pasture-mowed	21	19	90.48%
8	Hay-windrowed	363	350	96.42%
9	Oats	12	11	91.67%
10	Soybeans-notill	729	608	83.40%
11	Soybeans-min	1829	1587	86.77%
12	Soybeans-clean	457	366	80.09%
13	Wheat	159	157	98.74%
14	Woods	954	930	97.48%
15	Building-trees-	301	176	58.47%
16	Stone-steel	67	58	86.57%
/	Overall classification accuracy	/	/	86.93%

**Table 9:** Confusion matrix for maximum overlap pooling CNN classification used Indian Pines dataset.

Category	1	2	3	4	5	6	7	8	9	10	11	12	13	14	15	16
1	22	0	0	0	1	0	0	11	0	0	1	1	0	0	0	0
2	0	844	21	7	1	0	0	0	3	54	103	9	0	0	1	0
3	0	14	475	36	0	0	0	0	1	6	63	15	0	0	1	0
4	0	4	11	136	0	1	0	0	2	0	13	5	0	0	1	0
5	1	0	0	1	321	8	0	0	0	0	10	2	0	3	4	0
6	0	0	0	2	0	521	0	0	0	0	2	0	0	3	14	0
7	0	0	0	0	0	0	21	0	0	0	0	0	0	0	0	0
8	1	0	0	0	00	0	0	360	0	0	1	0	0	0	1	0
9	0	0	0	0	00	0	0	0	12	0	0	0	0	0	0	0
10	0	24	3	3	3	2	0	0	0	619	70	4	0	0	1	0
11	0	40	46	4	2	2	1	0	0	56	1661	8	0	0	9	0
12	0	9	19	4	4	1	0	0	0	2	27	387	0	0	3	1
13	0	0	0	0	0	0	0	0	1	0	1	0	156	0	1	0
14	0	0	0	0	6	1	0	0	0	0	0	0	1	918	28	0
15	0	0	0	0	8	21	0	0	4	0	2	0	4	63	198	1
16	0	0	0	0	1	0	0	0	0	0	4	0	0	0	1	61

**Table 10:** Statistical tables for maximum overlap pooling CNN classification charting accuracy used Indian Pines dataset.

No.	Ground Category	Total Number of Pixels	Correct Classification	Classification Accuracy
1	Alfalfa	36	22	61.11%
2	Corn-notill	1043	844	80.92%
3	Corn-min	611	475	77.74%
4	Corn	173	136	78.61%
5	Grass/Pasture	350	321	91.71%
6	Grass/Trees	542	521	96.13%
7	Pasture-mowed	21	21	100.00%
8	Hay-windrowed	363	360	99.17%
9	Oats	12	12	100.00%
10	Soybeans-notill	729	619	84.91%
11	Soybeans-min	1829	1661	90.81%
12	Soybeans-clean	457	387	84.68%
13	Wheat	159	156	98.11%
14	Woods	954	918	96.23%
15	Building-trees	301	198	65.78%
16	Stone-steel	67	61	91.04%
/	Overall classification accuracy	/	/	87.78%

**Table 11:** Confusion matrix for traditional CNN classification used Salinas dataset.

Category	1	2	3	4	5	6	7	8	9	10	11	12	13	14	15	16
1	1470	15	0	0	0	0	0	0	0	0	0	0	0	0	0	0
2	1	2789	0	0	0	0	0	1	0	0	0	0	1	0	0	1
3	0	0	1458	4	0	0	0	0	0	0	0	0	0	0	0	0
4	0	0	1	1048	2	0	0	0	0	0	0	0	0	0	0	0
5	0	0	99	10	1896	0	0	0	2	0	0	0	0	0	0	0
6	0	0	0	0	1	2981	0	0	0	0	0	0	0	0	0	0
7	0	1	0	0	0	0	2641	1	0	0	0	0	1	4	0	1
8	0	0	0	0	0	0	0	7540	1	25	0	0	0	5	873	1
9	0	0	0	0	0	0	0	0	4666	1	0	0	0	0	0	0
10	0	0	3	1	3	0	0	16	27	2389	2	4	1	13	0	6
11	0	0	0	0	0	0	0	0	0	0	805	0	0	0	0	0
12	0	0	0	0	0	0	0	0	0	0	2	1430	0	2	0	0
13	0	0	0	0	0	0	0	0	0	0	0	0	703	2	0	0
14	0	0	0	0	0	0	0	1	1	2	0	0	13	815	0	0
15	0	0	2	0	1	0	1	1416	0	20	0	0	0	0	4021	1
16	0	4	0	0	0	0	0	0	0	1	0	0	0	0	0	1349

**Table 12:** Statistics of traditional CNN classification chart accuracy used Salinas dataset.

No.	Ground Category	Total Number of Pixels	Correct Classification	Classification Accuracy
1	Brocoli_green_weeds_1	1485	1470	98.99%
2	Brocoli_green_weeds_2	2793	2789	99.86%
3	Fallow	1462	1458	99.73%
4	Fallow_rough_plow	1051	1048	99.71%
5	Fallow_smooth	2007	1896	94.47%
6	Stubble	2982	2981	99.97%
7	Celery	2649	2641	99.70%
8	Grapes_untrained	8445	7540	89.28%
9	Soil_vinyard_develop	4667	4666	99.98%
10	Corn_sensced_green_weeds	2465	2389	96.92%
11	Lettuce_romaine_4wk	805	805	100%
12	Lettuce_romaine_5wk	1434	1430	99.72%
13	Lettuce_romaine_6wk	705	703	99.72%
14	Lettuce_romaine_7wk	832	815	97.96%
15	Vinyard_untrained	5462	4021	73.62%
16	Vinyard_vertical_trellis	1354	1349	99.63%
/	Overall classification accuracy	/	/	93.60%

**Table 13:** Confusion matrix for maximum overlap pooling CNN classification used Salinas dataset.

Category	1	2	3	4	5	6	7	8	9	10	11	12	13	14	15	16
1	1479	4	0	0	0	0	0	0	0	0	0	0	0	0	0	2
2	0	2792	0	0	0	0	0	0	0	0	0	0	0	0	0	1
3	0	0	1546	0	2	0	0	0	0	4	0	0	0	0	0	0
4	0	0	0	1046	5	0	0	0	0	0	0	0	0	0	0	0
5	0	0	1	8	1997	0	0	0	0	0	1	0	0	0	0	0
6	0	1	0	0	1	2980	0	0	0	0	0	0	0	0	0	0
7	0	1	0	0	0	1	2642	0	0	0	0	0	0	3	0	1
8	0	0	0	0	0	0	0	7576	1	7	0	0	0	0	861	0
9	0	0	0	0	0	0	0	0	4666	1	0	0	0	0	0	0
10	0	0	0	1	1	2	0	19	24	2399	1	2	0	8	3	5
11	0	0	0	0	0	0	0	0	1	0	802	2	0	0	0	0
12	0	0	0	0	0	0	0	0	0	0	0	1432	2	0	0	0
13	0	0	0	0	0	0	0	0	0	0	0	0	703	2	0	0
14	0	0	0	0	0	0	0	1	0	5	0	0	12	814	0	0
15	0	0	0	0	2	0	0	1069	0	4	0	0	0	0	4387	0
16	0	4	0	0	0	0	0	0	0	0	0	0	0	0	0	1350

**Table 14:** Statistical tables for maximum overlap pooling CNN classification charting accuracy used Salinas dataset.

No.	Ground Category	Total Number of Pixels	Correct Classification	Classification Accuracy
1	Brocoli_green_weeds_1	1485	1479	99.60%
2	Brocoli_green_weeds_2	2793	2792	99.96%
3	Fallow	1552	1546	99.61%
4	Fallow_rough_plow	1051	1046	99.52%
5	Fallow_smooth	2007	1997	99.50%
6	Stubble	2982	2980	99.93%
7	Celery	2648	2642	99.77%
8	Grapes_untrained	8445	7576	89.71%
9	Soil_vinyard_develop	4667	4666	99.98%
10	Corn_sensced_green_weeds	2465	2399	97.32%
11	Lettuce_romaine_4wk	805	802	99.63%
12	Lettuce_romaine_5wk	1434	1432	99.86%
13	Lettuce_romaine_6wk	705	703	99.72%
14	Lettuce_romaine_7wk	832	814	97.84%
15	Vinyard_untrained	5462	4387	80.32%
16	Vinyard_vertical_trellis	1354	1350	99.70%
/	Overall classification accuracy	/	/	94.90%
